# E-cigarettes versus NRT for smoking reduction or cessation in people with mental illness: secondary analysis of data from the ASCEND trial

**DOI:** 10.1186/s12971-015-0030-2

**Published:** 2015-03-24

**Authors:** Brigid O’Brien, Oliver Knight-West, Natalie Walker, Varsha Parag, Christopher Bullen

**Affiliations:** National Institute for Health Innovation, School of Population Health, The University of Auckland, Auckland, New Zealand

**Keywords:** Electronic cigarette/e-cigarette, Tobacco, Smoking cessation, Mental illness, Nicotine, Harm reduction

## Abstract

**Background:**

People with mental illness have higher rates of smoking than the general population and are at greater risk of smoking-related death and disability. In smokers from the general population, electronic cigarettes (e-cigarettes) have been shown to have a similar effect on quit rates as nicotine replacement therapy, but little is known about their effect in smokers with mental illness.

**Methods:**

Secondary analysis of data from the ASCEND trial involving 657 dependent adult smokers motivated to quit, randomised to 16 mg nicotine e-cigarette, 21 mg nicotine patch, or 0 mg nicotine e-cigarette, with minimal behavioural support. Using self-reported medication use and the Anatomical Therapeutic Chemical Classification System, we identified 86 participants with mental illness and analysed their cessation and smoking reduction outcomes.

**Results:**

For e-cigarettes alone, and all interventions pooled, there was no statistically significant difference in biochemically verified quit rates at six months between participants with and without mental illness, nor in smoking reduction, adverse events, treatment compliance, or acceptability. Rates of relapse to smoking were higher in participants with mental illness. Among this group, differences between treatments were not statistically significant for cessation (patch 14% [5/35], 16 mg e-cigarette 5% [2/39], 0 mg e-cigarette 0% [0/12], p = 0.245), adverse events or relapse rates. However, e-cigarette users had higher levels of smoking reduction, treatment compliance, and acceptability.

**Conclusions:**

The use of e-cigarettes for quitting appears to be equally effective, safe, and acceptable for people with and without mental illness. For people with mental illness, e-cigarettes may be as effective and safe as patches, yet more acceptable, and associated with greater smoking reduction.

**Trial registration:**

Australian New Zealand Clinical trials Registry, number: ACTRN12610000866000.

## Background

People affected by mental illness suffer excess morbidity and mortality, with much of this disparity attributable to cigarette smoking [1,2]. Such people are more cigarette dependent, smoke more heavily [3,4], are less likely to quit [4,5] and have a smoking prevalence several times higher than those without mental illness [6]. Because mental illness is common, affecting around a quarter of people in any year in developed countries [7-9], smoking in this population accounts for a large proportion of the smoking-related disease burden and associated economic costs for health systems [10]. Reducing smoking rates in people with mental illness is a public health priority.

People with mental illness are motivated to quit smoking [4,11], but comparatively few data exist on the efficacy of standard cessation interventions when used by this population because they are routinely excluded from clinical trials. The available evidence suggests bupropion is effective, but it is unclear for varenicline and mixed for nicotine replacement treatment (NRT) [2,12-17]. Despite a reduction in smoking prevalence across the general population of most developed countries, there has been little progress in boosting successful quit rates among smokers with mental illness [7,18,19]. An urgent need exists to increase the availability of cessation interventions that have appeal and address the high levels of cigarette addiction in this population [18].

Electronic cigarettes (e-cigarettes) are nicotine delivery devices that have promise for smoking cessation: two published randomised controlled trials (RCTs) involving first generation e-cigarettes with low nicotine delivery among general populations of smokers suggest modest effectiveness, similar to nicotine patches with minimal behavioural support [20,21]. A third trial involving ‘second generation’ e-cigarettes also supports these findings [22]. Little is known about e-cigarette use in people with mental illness. Only six studies have been reported to date: a small (n = 14) uncontrolled pilot study in patients with schizophrenia [23], three case reports of patients (n = 1-2) with affective or anxiety disorders [24-26], a US population-based survey (n = 10,041) [27], and a recently published cohort study of 956 smokers with severe mental illness participating in a smoking cessation trial that did *not* involve e-cigarettes [28]. The latter study found that 11% of participants used an e-cigarette during the study, and concluded that e-cigarette use was not associated with changes in smoking behaviour. However, it is not possible to draw such firm conclusions from the data because people who ‘used’ an e-cigarette once and never again were not differentiated from those who used them frequently and for several months. The other studies suggested e-cigarettes have cessation and harm reduction potential for people with mental illness, including in those who were not motivated to quit, or who had previously tried to quit with NRT but failed. The latter finding raises the possibility that e-cigarettes might have an advantage over NRT through their ability, over and above nicotine delivery, to provide a coping mechanism for conditioned smoking cues by replacing some of the rituals associated with smoking gestures [23].

In order to investigate the above hypothesis we examined data from the largest randomised controlled trial of e-cigarettes to date, the ASCEND trial, focussing on participants who stated they were taking mental illness-related medications, and therefore likely to have a mental illness. We hypothesised that 1) nicotine patches and e-cigarettes, individually and collectively, are equally as effective, safe and acceptable for smoking cessation and reduction for people with or without mental illness, and that 2) e-cigarettes are more effective and acceptable than nicotine patches for smoking cessation and reduction in people with mental illness.

## Methods

The ASCEND trial protocol and main findings have been described in detail elsewhere [21,29]. In brief, 657 dependent smokers aged ≥18 years, who were motivated to quit, were recruited from the community in Auckland, New Zealand (NZ), and randomised in a 4:4:1 ratio to 16 mg nicotine e-cigarettes (*ad libitum* use), 21 mg nicotine patches (one daily) or 0 mg e-cigarettes (*ad libitum* use), from one week before until 12 weeks after the nominated quit day. Low intensity behavioural support was offered via voluntary telephone counselling. Smokers with poorly controlled psychiatric disorders or chemical dependency other than nicotine were excluded. The Northern X Regional Ethics Committee of New Zealand approved the study (Number NTX/10/11/111); the Standing Committee on Therapeutic Trials approved the nicotine electronic cigarettes for research. All study procedures were conducted in accordance with the Declaration of Helsinki.

Participants were assessed by telephone at baseline, quit date (one week post-baseline), one, three and six months post quit-date. Concomitant medication use was assessed at each time-point. The Anatomical Therapeutic Chemical (ATC) Classification System was used to identify participants taking medications related to mental illness [30]. This system classifies drugs into groups according to system on which they act and their therapeutic, pharmacological and chemical properties [30]. ATC codes indicating mental illness include: antidepressants, psychostimulants, antipsychotics, anxiolytics, hypnotics/sedatives and drugs for addictive disorders. Participants were divided into two major groups; ‘mental illness participants’ (those who reported use of ≥1 of the medications associated with mental illness), and ‘non-mental illness participants’ (no reported use of any medications associated with mental illness).

Baseline measures comprised demographic and smoking-related variables, including nicotine dependence [31], the Glover-Nilsson smoking behavioural questionnaire [GN-SBQ] [32], motivation to quit measured on a scale of 1–5 (where 1 is very low and 5 is very high), and stage of addiction measured using the autonomy over smoking scale (AUTOS) [33]. The primary outcome was continuous smoking abstinence six months after quit day, verified by an exhaled breath carbon monoxide measurement of <10 ppm using a Bedfont Micro Smokerlyzer. Secondary outcomes included rate of smoking relapse (date of return to regular daily smoking), reduction in cigarettes smoked per day (CPD) in those who did not quit, treatment compliance (continuing use of treatment at three months), adverse events and acceptability measures (i.e. recommendation of product to friend; stopping use of product due to dislike).

Analyses were performed using SAS version 9.3. All tests were two-tailed, with significance set at 5%. Continuous outcomes were compared using t-tests, and binary outcomes were compared using chi-squared tests (or Fisher’s Exact test if cell counts were <5). Primary outcome analyses were undertaken on an intention-to-treat basis whereby participants with missing smoking status were assumed to be smoking. The primary outcome was adjusted for mental illness status (as defined above) using logistic regression.

## Results

Overall 86 (13%) of the 657 participants reported using ≥1 medication associated with mental illness and 571 (87%) reported no such medication use. Of the 86 mental illness participants, 39 were allocated to 16 mg e-cigarettes, 35 to nicotine patches and 12 to 0 mg e-cigarettes. Three-quarters (74%, 64/86) reported using antidepressants, 28% (24/86) antipsychotics, 14% (12/86) hypnotics/sedatives, 9% (8/86) anxiolytics, and 1% (3/86) addictive disorder medications.

Participants categorised as having a mental illness had a mean age of 44 years (SD = 12), mean smoking initiation age of 15 years (SD = 4), 66% (57) were female, almost half (44%, 38) had <12 years school education and half (43) had made a quit attempt in the previous year. At baseline, participants with and without mental illness were similar with respect to age, gender, education, previous quit attempts, behavioural dependence, motivation to quit and stage of addiction . Compared to participants without mental illness, those with mental illness were less likely to be Māori (indigenous New Zealanders) (8% [7/86] vs 36% [206/571, p < 0.001) or live with other smokers (40% [34/86] vs 54% [308/571], p = 0.012), and more likely to be nicotine dependent (mean Fagerstrom score 6.3 versus 5.4, p < 0.0001; highly dependent Fagerstrom score > 5: 70% vs 52%, p = 0.003) and smoke more CPD (mean 19.4 vs 17.8 respectively, p = 0.049). Baseline characteristics of participants with mental illness by intervention are shown in Table 1.

**Table 1:** **Baseline characteristics of mental illness participants by intervention**

**Characteristic**	**21 mg nicotine patch (n = 35)**	**16 mg e-cigarette (n = 39)**	**0 mg e-cigarette (n = 12)**
Mean age	41 (11)	46 (11)	46 (14)
Female gender	69% (24)	67% (26)	59% (7)
Mean Fagerstrom score	6.5 (2.0)	6.6 (1.7)	5.1 (2.4)
Reported antidepressant use	69% (24)	77% (30)	83% (10)
Reported antipsychotic use	29% (10)	23% (9)	42% (5)
Reported anxiolytic use	6% (2)	13% (5)	8% (1)
Reported hypnosedative use	9% (3)	15% (6)	25% (3)
Reported drugs for addictive disorders use	3% (1)	3% (1)	8% (1)

Data are mean (SD) or % (n).

### Comparison of outcomes for participants with and without mental illness

The pooled results across the three interventions for participants with mental illness compared with those without mental illness are shown in Table 2. No significant difference between the two groups was noted, except for a higher relapse rate in mental illness participants. The primary outcome remained unchanged after adjusting for mental illness. Forty-four adverse events occurred amongst 35 mental illness participants compared with 248 events amongst 194 non-mental illness participants. A single psychiatric adverse event occurred in the mental illness group: a depressive episode that was not considered study-related. The only study-related adverse event in this group was a sore throat in a person allocated 16 mg e-cigarettes.

**Table 2:** **Comparison of outcomes for participants with and without mental illness displaying both pooled and intervention level results for the three interventions (21 mg nicotine patch, 16 mg e-cigarette, 0 mg e-cigarette)**

**Outcome**	**Intervention**	**Mental Illness (n = 86, 13%) patch n = 35, 16 mg e-cigarette n = 39, 0 mg e-cigarette n = 12**	**No Mental Illness (n = 571, 87%) patch n = 260 , 16 mg e-cigarette n = 250, 0 mg e-cigarette n = 61**	**Difference (p value)**
**Biochemically verified continuous abstinence at six months % (n)**	All interventions pooled	8% (7)	6% (34)	0.435
	21 mg nicotine patch	14% (5)	5% (12)	0.038^a^
	16 mg e-cigarette	5% (2)	7% (19)	0.750^a^
	0 mg e-cigarette	0% (0)	5% (3)	-
**Relapse rate at six months % (n)**	All interventions pooled	79% (68)	67% (380)	0.020
	21 mg nicotine patch	71% (25)	67% (175)	0.931
	16 mg e-cigarette	85% (33)	66% (164)	<0.0001
	0 mg e-cigarette	83% (10)	67% (41)	0.239
**Mean reduction in CPD from baseline to six months in those that did not quit Mean (SD)**	All interventions pooled	7.7 (6.7)	8.4 (7)	0.508
	21 mg nicotine patch	5.7 (6.3)	7.4 (7)	0.299
	16 mg e-cigarette	9.9 (7)	9.4 (7.1)	0.743
	0 mg e-cigarette	4.7 (3.5)	8.3 (5.9)	0.129^b^
**Percentage reduction in CPD from baseline to six months in those that did not quit Mean (SD)**	All interventions pooled	40% (30%)	46% (33%)	0.154
	21 mg nicotine patch	29% (30%)	41% (35%)	0.147
	16 mg e-cigarette	49% (28%)	51% (31%)	0.660
	0 mg e-cigarette	31% (26%)	47% (28%)	0.245^b^
**Treatment compliance at three months % (n)**	All interventions pooled	39% (30)	37% (167)	0.757
	21 mg nicotine patch	20% (6)	18% (34)	0.752
	16 mg e-cigarette	53% (19)	51% (107)	0.861
	0 mg e-cigarette	46% (5)	54% (26)	0.741
**Adverse events rate (events/person month)**	All interventions pooled	0.078	0.084	0.666 (IRR 0.93, 95% CI 0.68-1.28)
	16 mg e-cigarette and 0 mg e-cigarette combined	0.05	0.05	0.592 (IRR 0.89, 95% CI 0.59-1.35)
**Acceptability of intervention at six months**				
*‘Would recommend to a friend’* **% (n)**	All interventions pooled	65% (49)	72% (313)	0.207
	21 mg nicotine patch	37% (11)	63% (19)	0.122
	16 mg e-cigarette	83% (30)	85% (175)	0.752
	0 mg e-cigarette	80% (8)	89% (42)	0.594^a^
*‘Stopped as didn’t like it ’* **% (n)**	All interventions pooled	34% (21/62)	32% (108/343)	0.711
	21 mg nicotine patch	41% (12/29)	41% (169/169)	0.956
	16 mg e-cigarette	29% (7/24)	23% (33/146)	0.482
	0 mg e-cigarette	22% (2/9)	21% (6/28)	1.000^a^

CPD = cigarettes per day smoked, SD = standard deviation, IRR = Incidence rate ratio, CI = confidence interval, ^a^Fishers Exact test, ^b^Mann-Whitney test.

Similar results were found when we analysed the data by treatment allocation (Table 2). For participants allocated to e-cigarettes there were no significant differences in primary or secondary outcomes between those with and without mental illness. The exception was smoking relapse which occurred at a higher rate in mental illness participants (Table 2). For participants allocated the nicotine patch no significant differences in outcomes were found between those with and without mental illness, with one exception: the six month quit rate was higher in participants with mental illness compared to those without mental illness (14% [5/35] vs 5% [12/260] respectively, p = 0.038).

### Comparison of interventions for mental illness participants

There were no significant differences in quit rates or relapse rates for participants with mental illness randomised to each of the three interventions (Table 3). Adverse event counts relative to the number of participants were similar (these were not subject to statistical testing due to small numbers). No serious study-related adverse events were noted in any group. For smoking reduction, compliance, and acceptability 16 mg e-cigarettes outperformed nicotine patches. No significant difference was detected between 16 mg and 0 mg e-cigarettes for any outcome tested. Among mental illness participants allocated 16 mg e-cigarettes, approximately half (53%) liked their tactile, cigarette-like qualities, sensory familiarity, perceived health benefits, taste and ease of use.

**Table 3:** **Comparison of outcomes for mental illness participants who used 16 mg nicotine e-cigarettes, 0 mg e-cigarettes and 21 mg nicotine patches**

**Outcome**	**21 mg nicotine patch (n = 35, 40%)**	**16 mg nicotine e-cigarette (n = 39, 45%)**	**0 mg nicotine e-cigarette (n =12, 14%)**	**Difference (p-value)**
**Biochemically verified continuous abstinence at six months % (n)**	14% (5)	5% (2)	0	0.245 (patch vs. 16 mg e-cig)^a^
				- (16 mg vs. 0 mg e-cig)
				0.115 (patch vs. combined e-cig)^a^
**Relapse rate at six months % (n)**	71% (25)	85% (33)	83% (10)	0.169 (patch vs. 16 mg e-cig)
				1.000 (16 mg vs. 0 mg e-cig)
				0.149 (patch vs. combined e-cig)
**Mean reduction in CPD from baseline to six months in those that did not quit Mean (SD)**	5.7 (6.3)	9.9 (7)	4.7 (3.5)	0.035 (patch vs. 16 mg e-cig)
				0.068 (16 mg vs. 0 mg e-cig)
				0.083 (patch vs. combined e-cig
**Percentage reduction in CPD from baseline to six months in those that did not quit Mean (SD)**	29% (30%)	49% (30%)	31% (30%)	0.025 (patch vs. 16 mg e-cig)
				0.153 (16 mg vs. 0 mg e-cig)
				0.049 (patch vs. combined e-cig)
**Treatment compliance at three months % (n)**	20% (6)	53% (19)	46% (5)	0.006 (patch vs. 16 mg e-cig)
				0.670 (16 mg vs. 0 mg e-cig)
				0.006 (patch vs. combined e-cig)
**Adverse events**	17 (in 16 people)	22 (in 15 people)	5 (in 4 people)	-
**Acceptability of intervention at six months**				
*‘Would recommend to a friend’* **% (n)**	37% (11)	83% (30)	80% (8)	<0.001 (patch vs. 16 mg e-cig)
				1.000a (16 mg vs. 0 mg e-cig)
				<0.001 (patch vs. combined e-cig)
*‘Stopped as didn’t like it’* **% (n)**	41% (12/29)	29% (7/24)	22% (2/9)	0.356 (patch vs. 16 mg e-cig)
				1.000a (16 mg vs. 0 mg e-cig)
				0.242 (patch vs. combined e-cig)

CPD = cigarettes per day smoked, SD = standard deviation, ^a^Fishers Exact test.

## Discussion

Our findings suggest e-cigarettes could be a useful option for smoking cessation or harm reduction in smokers with mental illness. Our finding that e-cigarettes may be as effective, safe and acceptable for smoking cessation and reduction in people with mental illness as in those without is consistent with literature on standard cessation treatments in mental illness populations [2]. These findings, when considered with the results of the main ASCEND trial (which demonstrated non-inferiority of e-cigarettes to NRT in a general population), also suggest a possible role for e-cigarettes in smokers with mental illness.

The absolute effectiveness of both NRT and e-cigarettes was low for smokers with or without mental illness. More intensive behavioural support would likely have improved the efficacy of all treatment arms across the entire sample, and is an especially important component of treatment for smokers with mental illness [34]. The extent of smoking reduction among participants without mental illness did not differ between treatments. However, at six months smokers with mental illness who had been allocated a nicotine e-cigarette smoked significantly fewer cigarettes than those allocated to patch or 0 mg e-cigarette. This potential for harm reduction in this population warrants further exploration, and aligns with research recommendations from the UK’s National Institute for Health and Clinical Excellence [35].

Among people with mental illness, e-cigarettes appeared to have a similar safety profile to nicotine patch, yet were generally more acceptable and associated with greater compliance. These findings align with the general population outcomes of the main trial. Many of the observed advantages of e-cigarettes over patch were evident irrespective of whether nicotine was present, suggesting that some of the benefit conferred is due to the tactile properties of the e-cigarette simulating the behavioural and sensory aspects of smoking. The absence of any difference between patch and e-cigarettes for quit rates and relapse rates is difficult to interpret given the small numbers. The reason for the higher quit rate observed for patch users with mental illness compared to those without is unknown, but may be a chance finding.

Limitations of the trial have been discussed in the original publication [21], including issues associated with the e-cigarettes used in the trial (e.g. variable nicotine content and delivery, and battery failure). Further limitations specific to these analyses should also be acknowledged, and indicate findings need to be interpreted with caution and considered exploratory. First, the analyses presented were of secondary data and post-hoc, and multiple tests were undertaken thereby increasing the chances of a type 1 error. Second, the analyses involved a small sample size and therefore there was limited power to detect subgroup differences. Finally, the generalisability of the findings to the population of people with mental illness is limited by our use of a proxy measure for a diagnosis of mental illness and the exclusion of people with uncontrolled psychiatric or current chemical dependence from the trial. It is possible that medication use was under-reported, some mental illnesses may have been undiagnosed and therefore unmedicated, and some medications associated with mental illness could have been prescribed instead for pain or sleep disorders. On balance we believe our sample probably best represents those with moderate mental illness, while excluding those with very severe or milder (unmedicated) mental illness. It is also possible that a small proportion taking medication for pain or sleep disorders were wrongly classified as having mental illness. In our study population 13% reported using one or more mental health medications, whereas in a survey of 2,299 NZ smokers between 2007–09 20% had ever had a mental illness diagnosis and 10% had a high probability of a depressive or anxiety disorder [36]. Notwithstanding the prevalence of mental illness is likely to be higher in more dependent smokers than the general population of smokers, our findings align with population estimates.

## Conclusions

Our findings suggest e-cigarettes are similarly effective, safe, and acceptable for smoking cessation and reduction in people with mental illness as those without; furthermore among people with mental illness they appear to be favoured over nicotine patches while yielding a greater decrease in cigarette consumption. To improve health outcomes in this priority group, new approaches to cessation support and harm reduction are urgently needed; our study suggests e-cigarettes warrant further investigation in this regard.

## Abbreviations

ATC: Anatomical Therapeutic Chemical
AUTOS: Autonomy over smoking scale
E-cigarettes: Electronic cigarettes
GN-SBQ: Glover-Nilsson smoking behavioural questionnaire
NRT: Nicotine replacement therapy
NZ: New Zealand
RCT: Randomised controlled trial
